# Applications of robotic catheter ablation in congenital heart disease

**DOI:** 10.3389/fped.2026.1807790

**Published:** 2026-04-10

**Authors:** Paul Khairy, Sabine Ernst

**Affiliations:** 1 Electrophysiology Service and Adult Congenital Heart Disease Centre, Montreal Heart Institute, Université de Montréal, Montreal, QC, Canada; 2 Electrophysiology Service, Royal Brompton Hospital, London, United Kingdom

**Keywords:** arrhythmia (any), catheter ablation, congenital heart disease, robotic interventions, robotic magnetic-guided navigation

## Abstract

Robotic catheter ablation has evolved from a niche technology into a practical platform for treating arrhythmias in patients with congenital heart disease, a population in whom altered vascular routes, surgically created baffles and conduits, chamber enlargement, and prosthetic material frequently render catheter access and stability dominant procedural challenges. Robotic magnetic navigation, the principal robotic system used in congenital heart disease, combines a highly flexible magnet-tipped catheter with externally controlled magnetic fields to enable precise catheter orientation and stable contact in the context of challenging vascular access and complex anatomies. Although the evidence base for robotic ablation in congenital heart disease remains largely observational, contemporary pooled analyses, case series, and focused reports consistently support its feasibility, safety, and high acute effectiveness. Beyond procedural success, these data suggest meaningful advantages in selected domains, including reduced radiation exposure and less operator fatigue during prolonged, high-complexity cases. Importantly, robotic navigation may also serve as an enabling technology in anatomies that are otherwise difficult to access or associated with increased procedural risk using conventional manual approaches. This perspective synthesizes the current literature, proposes practical criteria for selecting cases with congenital heart disease in which robotic ablation should be considered early, and outlines a forward-looking research and implementation agenda emphasizing anatomy-driven indications, and standardized workflows.

## Introduction

Arrhythmias are a leading cause of morbidity and hospitalizations in patients with congenital heart disease. They typically occur years after surgery, are often macroreentrant and scar-related, and are associated with adverse clinical outcomes (1, 2). For many patients with congenital heart disease, success of catheter ablation is limited less by uncertainty regarding mechanism than by the ability to reach the arrhythmogenic chamber, maintain stable catheter contact, and deliver contiguous lesions in the setting of distorted vasculature and surgically altered pathways (3, 4). Robotic magnetic navigation (RMN), the principle robotic system used in congenital heart disease, was developed, in part, to address these limitations. Large permanent external magnets generate adjustable fields that orient a soft magnetic catheter tip, enabling robotic vector-based steering and remotely controlled catheter movements (5). Early clinical experience established the feasibility of fully remote-controlled robotic mapping and ablation (6). With RMN, steering is achieved through magnetic vectors rather than torque transmission along a stiff shaft, which allows navigation through tortuous or convoluted pathways in a controlled fashion. In complex congenital anatomy, these features expand the range of substrates that can be treated percutaneously by enabling access to the target region with more dependable lesion formation.

This perspective is intended for congenital cardiologist and electrophysiologists and focuses on the applications of RMN in congenital heart disease. It summarizes supporting literature, distinguishes where evidence remains sparse, and offers a pragmatic framework for clinical adoption and future investigation.

## Evidence base and what it supports today

The most comprehensive synthesis of RMN-guided ablation in congenital heart disease is a systematic review and pooled analysis by Vô and colleagues (7). Across 24 non-overlapping reports including 167 patients with congenital heart disease undergoing 202 RMN procedures for 260 arrhythmias, the most frequent targets were macroreentrant atrial tachycardias (7). Pooled estimates demonstrated high acute success {89.2% [95% confidence interval (77.8%, 97.4%)]}, favorable recurrence-free follow-up {84.5% [95% confidence interval (72.5%, 94.0%)]} over roughly two years, low complication rates, and short fluoroscopy times, with no reported complications attributed directly to RMN technology (7). While the included studies were heterogeneous in lesion types, arrhythmia mechanisms, and local workflows, the overall signal was consistent: in expert hands, RMN is a safe and effective tool for ablation across diverse complex congenital substrates.

To our knowledge, comparative data evaluating RMN vs. manual ablation in congenital heart disease is limited to one retrospective study (8). In a 1:3 propensity-matched population of 63 patients with surgically corrected congenital heart disease undergoing RMN vs. manual ablation for atrial arrhythmias, RMN was associated with lower fluoroscopy exposure, longer procedure duration, and similar complication rates. Freedom from atrial tachyarrhythmia at 36 months was greater with RMN (78.9% vs. 47.2%; hazard ratio 0.32).

These observations align with prior reviews emphasizing that RMN may be particularly suited to surgically altered anatomy and difficult catheter stability scenarios (9). More recent literature has shifted the emphasis from feasibility toward practical problem-solving in access-limited anatomies. RMN has been framed as a strategy to “overcome access challenges,” with descriptions of how distorted vascular pathways and post-surgical reconstructions constrain manual catheter maneuverability (3). It has been argued that patients with congenital heart disease are among the populations most likely to benefit from RMN precisely because its strengths address major limitations of standard manual ablation in this population (10).

**Figure 1:**
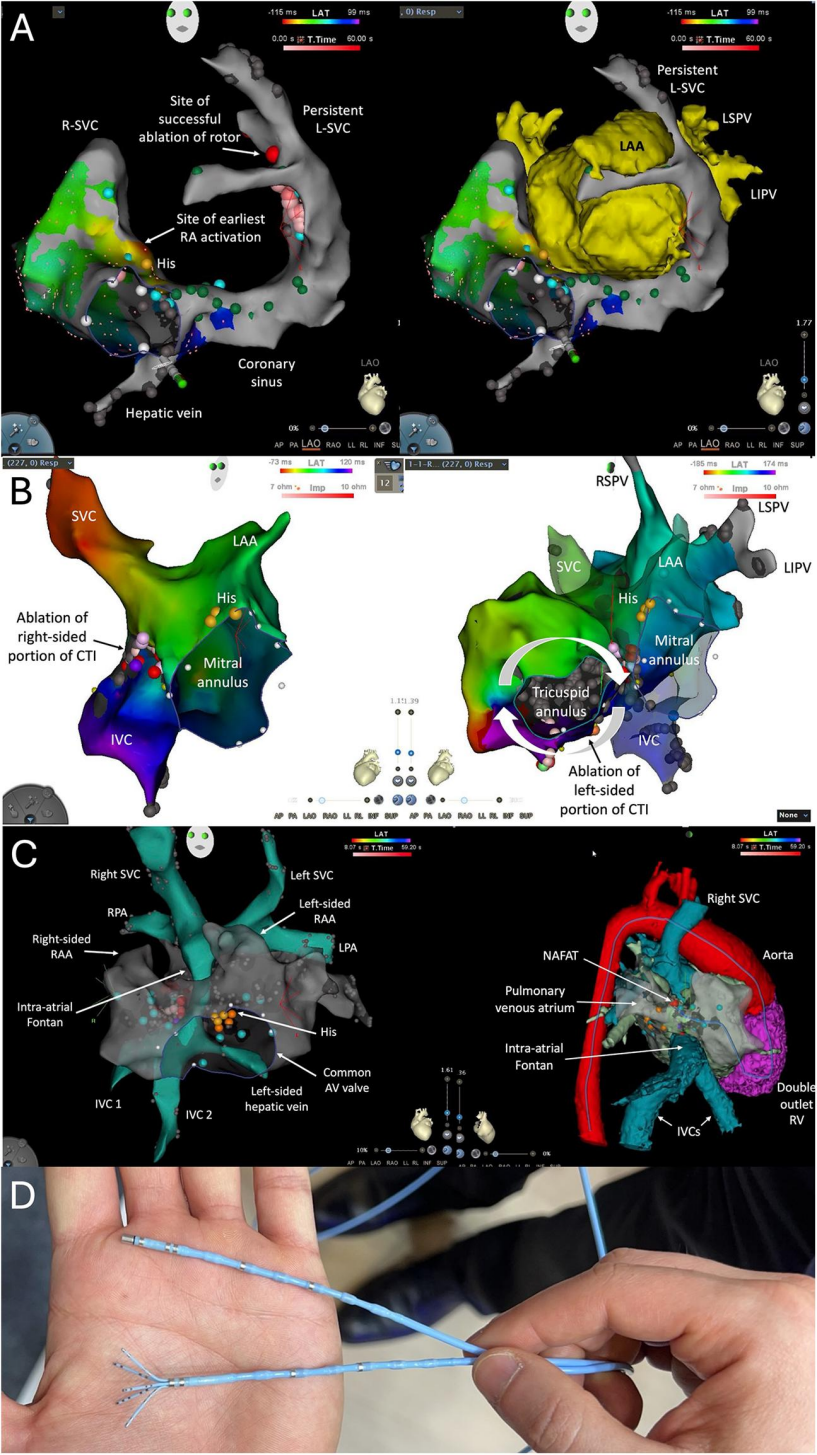
Examples of patients with congenital heart disease referred for RMN-guided ablation and new multipolar mapping catheters. Panel **(A)** depicts a patient with left atrial isomerism, abdominal situs solitus, interrupted inferior vena cava (IVC) with azygos vein continuation, and a persistent left superior vena cava (L-SVC) draining into a coronary sinus (CS). Catheter ablation of a rotor was performed, via access by the azygos vein, at the septal portion of the L-SVC, anterior to the left superior pulmonary vein (LSPV) and posterior to the left atrial appendage (LAA). LIPV denotes left inferior pulmonary vein. Panel **(B)** represents a patient with transposition of the great arteries and Mustard baffle with an occluded IVC inferior to the renal veins. The arrhythmia consisted of a biatrial clockwise cavotricuspid isthmus (CTI)-dependent circuit, with ablation of the right-sided component of the CTI performed in the systemic venous atrium by means of a right jugular venous approach and the left-sided portion in the pulmonary venous atrium using a retrograde aortic approach. RSPV denotes right superior pulmonary vein. Panel **(C)** shows the case of a patient with a double-outlet right ventricle (RV), right-atrial isomerism, common atrioventricular (AV) valve, bilateral SVCs and IVCs, and an intra-atrial Fontan. Catheter ablation of a non-automatic focal atrial tachycardia (NAFAT) was performed by a retrograde aortic approach. RAA denotes right atrial appendage; RPA, right pulmonary artery; LPA, left pulmonary artery. Shown in Panel **(D)** are new robotically navigated electrophysiology catheters, a 6-electrode saline-irrigated radiofrequency ablation catheter (MagBot, Everpace) and a “flower-shaped” 3D high-density mapping catheter (EASYStar, Everpace).

Nevertheless, existing data do not definitively establish comparative superiority of RMN over contemporary manual approaches across broad congenital heart disease populations. The literature remains dominated by observational series, often enriched for difficult anatomy or prior failed procedures, and outcomes likely reflect the intersection of technology with center expertise (3, 7, 9). The literature is also limited by small sample sizes, heterogeneity in congenital substrates and arrhythmia mechanisms, and potential referral bias toward more complex or previously failed cases. Moreover, outcomes are likely influenced by center-specific expertise and operator experience, which may limit generalizability. These factors underscore the need for cautious interpretation of pooled estimates and highlight the importance of prospective, multicenter studies with standardized endpoints. While it cannot be claimed that RMN “outperforms” manual ablation universally, it reliably enables safe and effective ablation in complex congenital heart disease settings and may offer incremental value when access, reach, and/or catheter stability are the limiting steps (Figure 1).

### Access as the defining problem in congenital ablation

In congenital heart disease, surgically created baffles, Fontan pathways, interrupted inferior vena cava, venous occlusions, and abnormal systemic venous return frequently constrain conventional access routes for mapping and ablation (10). RMN can be advantageous in these settings because catheter orientation is controlled magnetically at the tip, with fewer mechanical constraints imposed by catheter shaft stiffness. Even after multiple turns through tortuous or surgically altered pathways, the catheter can be steered with accuracy and precision. Clinical relevance of this principle is illustrated in case-based and technique-focused reports that highlight how unconventional trajectories can be executed when standard routes are otherwise constrained (11–15). Table 1 outlines practical contexts in congenital heart disease in which RMN may provide particular advantages.

**Table 1:** Congenital heart disease (CHD) scenarios in which RMN may provide procedural advantages.

CHD substrate/ patient group	Dominant procedural barrier	RMN advantage	Practical implication for workflow
Surgically altered atrial anatomy and baffles (e.g., atrial switch procedures)	Limited access to pulmonary venous atrium; sharp angulations and constrained working space	Vector-based steering enables controlled navigation after multiple turns and stable catheter orientation in complex geometries	Pre-procedural CT/MRI integration; early commitment to retrograde or alternative access strategies when required
Fontan circulation with atrial arrhythmias	Extensive atrial dilation; lack of transvenous access to the pulmonary venous atrium; fragile hemodynamics	Obviates need for higher-risk trans-conduit/trans-caval punctures to access pulmonary venous atrium; Stable magnetic orientation may improve mapping completeness and facilitate contiguous linear lesions	Early commitment to retrograde aortic access in absence of shunt; minimize remapping due to instability
Repaired tetralogy of Fallot with severe pulmonary regurgitation and RV dilation (including scar-related VT)	Difficulty maintaining stable contact for ventricular stimulation and catheter ablation in RVOT in the context of free pulmonary regurgitation and severely enlarged RV	Pull-force of magnetic field provides enhanced catheter stability for detailed mapping and ablation of the most common critical anatomical isthmuses involved in macroreentrant VT	Anticipate instability in context of severe PR and massive RV dilation; treat stability as a primary procedural objective
Markedly enlarged atria or ventricles in long-standing CHD	Extreme chamber enlargement leading to catheter “floating” and inconsistent contact	Magnetic steering allows precise vector alignment independent of shaft torque transmission, potentially improving point stability in large cavities	Use mapping density and electrogram consistency to confirm stability; adjust field vectors deliberately rather than relying on torque
Venous occlusion or need for alternative vascular access	Standard femoral access unavailable; unconventional routes required	RMN may preserve catheter controllability despite long, tortuous, or upper-extremity access pathways	Define access plan before puncture; escalate early if stability compromised
Complex CHD after failed manual ablation	Prior failure related to access or instability rather than irreducible mechanism	RMN may convert previously unstable mapping regions into treatable targets	Redesign trajectory and workflow rather than repeating manual approach

CT, computed tomography; MRI, magnetic resonance imaging; RV, right ventricle; RVOT, RV outflow tract; VT, ventricular tachycardia; PR, pulmonary regurgitation.

Ernst and colleagues reported the use of peripheral vascular access for ablation supported by RMN, demonstrating that non-traditional access routes can be paired with stable navigation when femoral access is not feasible (16). These reports highlight that the principal value of RMN in congenital heart disease lies in expanding feasible access options, rather than in incremental improvements to lesion delivery when access is already straightforward.

### Catheter stability, lesion quality, and congenital substrates

In congenital heart disease, catheter access, stability, and reach often determine not only whether ablation is feasible, but also how mapping can be performed. In access-limited anatomies, operators have historically resorted to point-by-point mapping rather than high-density mapping when only a single RMN-guided catheter could be advanced into the targeted chamber. That constraint is evolving with the introduction of RMN-guided high-density electrophysiology mapping catheters (Figure 1D). The Magic Sweep catheter (Stereotaxis, St. Louis, Missouri) is equipped with 20 electrodes arranged along its shaft. It received FDA clearance in July 2025. In addition, a multielectrode “flower-shaped” catheter (EASYStar, Everpace, Shanghai, China) has received CE-mark and can be integrated with a 3D electroanatomical mapping system (Coumbus, Everpace). Together with the emergence of an open RMN platform that supports integration with a broader range of mapping systems, these advances enable high-density mapping in chambers that are accessible only with RMN, potentially improving mapping resolution and procedural efficiency without requiring separate manually steered multipolar catheters.

In a study that quantified forces generated by magnetic navigation in a bench model, the average contact force was on the order of 6 g and increased substantially (capped at roughly the low-20 g range) when a long sheath was positioned at the chamber entrance (17). The practical implication is that RMN generates a predictable and bounded range of contact that is effective for catheter ablation and is associated with a low risk of cardiac perforation. Experimental work also supports the concept that RMN may reduce catheter tip displacement in the presence of simulated cardiac motion, which can influence lesion formation. In a myocardial phantom model with simulated wall motion, RMN produced larger lesion dimensions and volumes when compared with manual ablation, consistent with improved stability at the tissue interface (18). Complementary studies found that RMN-guided ablation of AV node reentrant tachycardia led to similar clinical outcomes as manual ablation, but with lesser release of high-sensitivity troponin T, corresponding to less unintended myocardial damage (19).

A contact-quality feedback system feature was added to the RMN-guided ablation system, i.e., the e-Contact Module, to address limitations about monitoring catheter-tissue interactions (20). Contact feedback has been associated with improved arrhythmia-free survival at one year post ablation (21). More recent bench and early translational work continues to refine the determinants of magnetic-guided contact force and its variability, including the influence of magnetic field strength, vector orientation, catheter extension, and sheath configuration, and this is especially pertinent as newer-generation RMN catheters are introduced (22). For congenital electrophysiology, the practical message is that stability and contact are not abstract advantages and that RMN can provide bounded and configurable contact forces with the potential to improve the durability of ablation lines in enlarged or access-constrained chambers.

### Implementation in congenital electrophysiology programs

RMN is best viewed as a system rather than a catheter. Laboratory configuration, integration with electroanatomic mapping and 3D imaging, staffing, and communication between the operator and bedside team are important elements of the workflow. A position paper from the Society for Cardiac Robotic Navigation synthesized best practices for RMN-guided ablation workflows, with recommendations that are directly relevant to congenital programs adopting RMN (23). The value of such consensus guidance is to help standardize processes, which is particularly important when rare anatomies and low procedural volumes limit experiential learning. Congenital programs require additional layers of planning beyond general RMN workflow. Pre-procedural review of operative notes and three-dimensional imaging is essential to map venous pathways, baffles, and chamber connections, and to decide up front whether a case is likely to require alternative vascular access or a retrograde aortic trajectory (24).

## Discussion and future directions

The next phase of RMN-guided ablation in congenital heart disease should move from broad feasibility to evidence-based anatomy-driven indications based on evidence demonstrating superiority. The current literature supports that RMN can be safe and effective in complex congenital substrates, with low fluoroscopy exposure and low complication rates in expert centers (3, 7, 9). What remains unresolved is how to deploy RMN on a broader scale most efficiently, given its capital cost, infrastructure needs, and training requirements. A practical research agenda should focus on identifying situations in which RMN changes procedural feasibility and patient-centered outcomes rather than simply replicating manual success rates. In congenital heart disease, outcomes that matter include successful access to the target chamber without escalation, recurrences that drive repeat interventions, and complications that shift the risk-to-benefit ratio. These endpoints align with congenital constraints and are more likely to reveal true incremental value than generic metrics alone.

Technological integration is likely to further magnify RMN's relevance. The congenital literature increasingly emphasizes pre-procedure imaging integration and access planning as central to RMN's utility (3). The logical next steps include standardized congenital segmentation workflows and integration of “digital anatomy” into mapping systems. These address uncertainties about where a catheter can be safely navigated and what tissue can be contacted.

From an implementation standpoint, RMN adoption in congenital heart disease should be linked to standardized workflows and to congenital-specific protocols that address imaging review, access planning, and contingency management (3, 23). RMN should be viewed as a platform that expands the range of treatable anatomies and may reduce risks associated with manual ablation in complex access scenarios. Our perspective is that RMN should be considered early when access and stability are expected to be dominant barriers, and that its evaluation should be grounded in congenital-specific endpoints that reflect feasibility, durability, and lifetime procedural burden. The evidence base has reached the point where thoughtful deployment is justified in congenital specialized centers, but the field needs collaborative prospective data to move from promising experience to evidence-based indications.

## Data Availability

The original contributions presented in the study are included in the article/Supplementary Material, further inquiries can be directed to the corresponding author.
